# The effects of dietary polyunsaturated fatty acids on miR-126 promoter DNA methylation status and VEGF protein expression in the colorectal cancer cells

**DOI:** 10.1186/s12263-018-0623-5

**Published:** 2018-12-18

**Authors:** Mostafa Moradi Sarabi, Seyed Abdollah Zahedi, Naser Pajouhi, Peyman Khosravi, Shahrokh Bagheri, Hassan Ahmadvand, Soroosh Shahryarhesami

**Affiliations:** 1 Department of Biochemistry and Genetics, Lorestan University of Medical Sciences, School of Medicine, Khorramabad, 381251698 Iran; 2 Department of Physiology, School of Medicine, Lorestan University of Medical Sciences, Khorramabad, Iran; 3 Razi Herbal Medicines Research Center, Lorestan University of Medical Sciences, Khorramabad, Iran; 4 Student Research Committee, Lorestan University of Medical Sciences, Khorramabad, Iran; 5 Functional Genome Analysis, German Cancer Research Center (DKFZ), Heidelberg, Germany

**Keywords:** Colorectal cancer, miRNA, DNA methylation, PUFA, Protein expression

## Abstract

**Background:**

There is increasing evidence indicating an aberrant expression of miRNAs in colorectal cancer (CRC) development. Growing evidence has suggested that polyunsaturated fatty acids (PUFAs) could modulate the remodeling of the epigenome. No study has yet been published to examine the direct effect of PUFA on the promoter methylation of miRNAs. This study aimed to examine the potential clinical application of PUFA on the promoter DNA methylation of miR-126 and its angiogenic target molecule (VEGF) in the CRC cells.

**Methods:**

We investigated the direct effect of 100 μM EPA, DHA, and LA for 24 h on promoter methylation status of miR-126 in a panel of five CRC cell lines (HCT116, HT29/219, Caco2, SW742, and LS180) by methylation-specific PCR (MSP). We also quantified the miR-126 and VEGF transcript expression levels in five CRC cell lines affected by PUFA by real-time PCR. Moreover, we analyzed the protein expression level of VEGF, as a target of miR-126, by western blotting assay.

**Results:**

MSP analysis showed extensive DNA methylation of the miR-126 promoter in all five CRC cell lines, and among all three PUFAs, only DHA completely demethylated the promoter of miR-126 in HCT116 and Caco2 cell lines. We found that only DHA significantly induces the expression level of miR-126 in HCT116 and Caco2 cell lines, respectively, by 20.1-fold and 1.68-fold (*p* < 0.05). Our finding indicates that the downregulation of VEGF protein level is also effectively observed only in DHA-treated HCT116 and Caco2 cells compared to control cells (*p* < 0.05).

**Conclusions:**

Our results provide evidence that *n*-3 PUFAs are able to modulate cellular miR-126 DNA methylation and inhibit VEGF expression level in a cell-type specific manner in colorectal cancer cells. DHA always showed higher efficacy than EPA and LA in our experiment. Overall, our results suggest a potential clinical application of *n*-3 PUFAs as anti-angiogenic agents in CRC therapy.

## Background

Polyunsaturated fatty acids (PUFA) including eicosapentaenoic acid (EPA, 20:5, *n*-3) and docosahexaenoic acid (DHA, 22:6, *n*-3) are a component of marine oils that have been involved in the prevention of obesity, cardiovascular disease, neurodegenerative diseases, and metabolic diseases such as diabetes mellitus [1–4]. There is also ample evidence indicating that *n*-3 PUFAs exert antineoplastic effects against different types of cancer and that PUFA supplementation specially reduces the incidence and prevention of colorectal cancer (CRC) [1, 5–7]. Many studies have demonstrated that PUFA consumption in both rodent models of CRC and humans results in an increased PUFAs content in tumors and colonic mucosa, respectively [8, 9]. Also, other studies have reported that PUFA distribution is associated with CRC prognosis and *n*-3 PUFAs inhibit the growth of xenograft tumors of human CRC cell lines in rodents [10, 11]. From a mechanistic perspective, it has been suggested that dietary PUFAs might suppress cancer cell growth through different possible mechanisms, including cell migration, apoptosis, angiogenesis, signaling pathways, and regulation of gene expression [12–14]. There is also conceivable evidence indicating that PUFAs could modulate the remodeling of the epigenome and might modulate cellular microRNA (miRNA) signatures [15–17]. miRNAs are the small single-strand noncoding RNAs of 20–25 nucleotides in length that have been implicated in the regulation of diverse cellular processes, including cell differentiation, migration, invasion, and even tumor angiogenesis [18, 19]. miRNAs regulate gene expression post-transcriptionally through base-pairing with 3′-untranslated regions (3′UTRs) of target mRNA and causing repress gene expression by either mRNA degradation or inhibiting its translation [20]. Emerging evidence suggests that in human cancers, more than hundreds of miRNAs are indeed regulated at different levels by different mechanisms, including epigenetic alteration as 50% of them are known to be methylated in a cancer-specific manner in more than 20 different tumor types [21, 22]. In addition, many studies have revealed aberrant expression of miRNAs via aberrant DNA methylation in CRC development [23, 24]. miR-126 is an important regulatory miRNA which contributes to tumor angiogenesis, which is known as angiomiRs [25]. miR-126 restoration plays a pivotal role as a tumor suppressor through inhibition of vascular endothelial growth factor (VEGF) that serves as an oncogenic gene in tumor invasion and angiogenesis [26–28]. Moreover, recent studies have reported downregulation of miR-126 in multiple cancer types including cervical, pancreatic, and gastric and especially CRC samples [26, 29–32]. The association between expression level and clinicopathological features of miR-126 in CRC tissues has indicated that miR-126 expression level is significantly correlated with tumor invasion, inflammation, and angiogenesis of colorectal carcinogenesis [31]. Furthermore, recent studies have shown that DNA methylation results in the epigenetic silencing of miR-126 in colorectal cancer [33]. Many studies have indicated that *n*-3 PUFAs inhibited tumor growth by preventing the decrease in genomic DNA methylation in the CRC rat models [13]. Moreover, our quite recent study indicated that PUFAs altered the global and cell-type specific DNA methylation in human CRC cells [34]. However, the precise mechanism by which dietary PUFAs mediate epigenetic modifications in human cells is not fully demonstrated, and to our best knowledge in scientific literature, no published studies have yet examined if PUFAs can directly affect the alteration promoter methylation of miRNAs. We thus hypothesize that PUFAs can influence miR-126 gene expression through modulating its promoter methylation. For this purpose, we investigated the direct effect of *n*-3 and *n*-6 PUFAs on promoter methylation status of epidermal growth factor-like domain 7 gene (EGFL7), the host gene of miR-126, and protein expression level of VEGF, as a well target of miR-126, in a panel of five well-characterized colorectal cancer cell lines.

## Material and methods

### Chemicals

All chemicals and reagents were purchased from Gibco-Invitrogen (Paisley, UK) and Sigma Aldrich (Gillingham, UK).

### PUFA supplementation

We prepared BSA/PUFA conjugates for application to cells as described by Svedberg et al. [35]. Briefly, a stock solution of each pure fatty acid eicosapentaenoic acid (EPA, 20:5, *n*-3), docosahexaenoic acid (DHA, 22:6, *n*-3), and linoleic acid (LA, 18:2, *n*-6) was prepared by dissolving the free fatty acid in 50% (*v*/*v*) ethanol and stored in aliquots at − 20 °C protected from light until ready for use. Fresh PUFAs were prepared from a stock solution before every experiment by diluting in cell culture media containing 10 μM of cell culture-grade fatty acid -free BSA (to provide a carrier) and 100 μM of each PUFA (FA:BSA 10:1 ratio). The mixture was incubated at 37 °C for 2 h while being shaken to conjugates BSA/PUFA.

### Cell lines and cell culture

In this study, the five human colorectal cancer cell lines (HCT116, HT29/219, SW742, Caco2, and LS180) were obtained from the National Cell Bank of Iran (NCBI, Pasteur Institute, Tehran). HCT116, HT29/219, and SW742 cells were grown in RPMI 1640, LS180, and Caco2. Cells were cultured in DMEM supplemented with 10% FBS, 2 mM Gln, 100 U/ml penicillin, and 100 μg/ml streptomycin in a humidified 5% CO_2_ atmosphere at 37 °C. For all experiments, cells were seeded into six-well plates at a density of 3.0 × 10^4^ cells and allowed to attach for 24 h. Then, the cells were treated with 100 μM BSA-complexed PUFAs for 24 h. BSA-only media were served as our reference. The Trypan blue exclusion assay was used as a criterion for viability.

### Genomic DNA preparation

Genomic DNA was extracted from cultured cells by the standard method of proteinase K digestion, phenol-chloroform extraction, and ethanol precipitation as described previously [36].

### Bisulfite modification of genomic DNA and methylation analysis

To study the effect of PUFAs on miR-126 promoter methylation, CRC cell lines were treated with a 100 μM of BSA-complexed PUFAs for 24 h. Then, the status of miR-126 promoter methylation in CRC cell lines was determined by methylation-specific PCR (MSP) method as previously described [37]. Briefly, CRC genomic DNA samples were treated with sodium bisulfite and then PCR amplified using primers specific for either the methylated and modified unmethylated promoter region of miR-126. The primers and PCR conditions for miR-126 MSP analysis are listed in Table 1. In all MSP reactions, DNA from normal leukocytes and universal human methylated DNA standards from Zymo Research (ZYMO Research, Freiburg, Germany) were used as unmethylated (negative) and methylated (positive) controls, respectively.

**Table 1:** Primer sequence and the annealing temperature used for methylation-specific PCR

Gene	Forward primer	Reverse primer	Annealing *T* ( °C)
miR-126	U: 5′-GTGGTGGTGGTGTGTGTGTGTTT-3′	5′-CTCAACCCAACCCAAACAACAACCA-3′	60
	M: 5′-GCGGCGCGTGCGCGTTT-3′	5′-CCAACCCGAACGACGACCG-3′	

### Quantitation of miR-126 and VEGF with real-time RT-PCR

Total RNA was extracted from colorectal cancer cell lines using the TriPure isolation reagent (Roche Applied Science, Germany) according to the manufacturer’s instructions. The quantity of purified RNA was analyzed spectrophotometrically (Nanodrop, USA), integrity was assessed on 2% formaldehyde containing 1.5% agarose, and purified RNA was stored at − 80 °C until use. Expression levels of mature miR-126 and reference gene (U6 snRNA) were analyzed by stem-loop quantitative real-time RT-PCR assay using SYBR Green-based analysis and Master Mix (ABI, UK). All reactions were carried out in triplicate using a Corrbet sequence detection system (Rotor gene 6000). The real-time PCR amplification reactions were performed under the following conditions: 95 °C for 15 min followed by 40 cycles of 95 °C for the 30s, annealing at 60 °C for 60 s, and a final extension at 72 °C for 5 min. The primers’ sequence and PCR conditions for VEGF quantitative PCR analysis are listed in Table 2. The relative expression levels were determined using the 2^−∆∆CT^ standard method [38].

**Table 2:** Primers’ sequence used for quantitative real-time RT-PCR

Gene	Forward primer	Reverse primer	Annealing *T* (°C)
VEGF	5′-TGCAGATTATGCGGATCAAACC-3′	5′-TGCATTCACATTTGTTGTGCTGTAG-3′	60
GAPDH	5′-CGACCACTTTGTCAAGCTCA-3′	5′-AGGGGTCTACATGGCAACTG-3′	60

### Expression analysis of VEGF as a miR-126 target gene by western blot

Cell lysates were prepared using cold lysis buffer containing 150 mM NaCl, 1% Igipal CA-630, 50 mM Tris (pH, 8), and complete protease inhibitor (S8820). Then, the lysates were centrifuged at 12000 rpm for 20 min at 4 °C. The protein assay of each supernatant was done by Bradford method using bovine serum albumin as a standard. A volume of each sample containing 40 μg of protein was mixed with loading buffer comprised of 4% SDS, 10% 2-mercaptoethanol, 20% glycerol, 0.004% bromophenol blue, and 125 mM Tris HCl (pH = 6.8). Each sample was then denatured by boiling for 5 min and loaded onto a 12% sodium dodecyl sulfate-polyacrylamide gel. The protein separation was achieved by electrophoresis in a buffer containing 25 mM Tris, 250 mM glycine, and 0.1% SDS (pH = 8.3). The separated proteins were transferred to a 0.2-μm PVDF membrane by transfer buffer containing 48 mM Tris, 39 mM glycine, and 20% methanol (pH = 8.3). The non-specific binding blocking was accomplished by incubation of PVDF membrane in Tris-buffered saline 0.1% Tween 20 (TBST), containing 5% *w*/*v* nonfat dry milk for 1 h at RT; thereafter, the membrane was incubated individually overnight at 4 °C with antibodies against VEGF (ab 46,154, 1/1000) and GAPDH (97,166, 1/5000) as a loading control. The PVDF membrane was incubated with HRP conjugated secondary antibody (7074 or 7076, 1/5000) for 1 h at RT, the blots were visualized by ECL kit (RPN 2235). Band densitometry was done by ImageJ software, and each VEGF density value was normalized to that of the corresponding GAPDH.

### Statistical analysis

SPSS 18 analytic software (SPSS, Inc., Chicago) and GraphPad Prism (Version 6.01) were performed for data analysis. All data from three independent experiments are presented as mean ± standard deviation (SD) and were analyzed using one-way ANOVA followed by Tukey’s multiple comparison tests. Differences with *p* value ≤ 0.05 were set as the level of significance.

## Results

### Impact of PUFA on promoter methylation of miR-126 in CRC cell lines

**Fig. 1:**
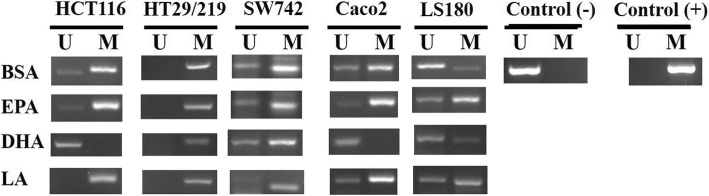
Representative MSP for promoter methylation analysis of EGFL7 (miR-126) in five CRC cell lines exposed to EPA, DHA, and LA. U, unmethylated genes; M, methylated genes; EPA, eicosapentaenoic acid; LA, linoleic acid; DHA, docosahexaenoic acid; BSA, bovine serum albumin

To study the impact of PUFA on DNA methylation, we analyzed the effect of PUFA on promoter methylation status of miR-126 in 5 CRC cell lines by MSP. Representative MSP of EGFL7 (miR-126) promoter methylation is shown in Fig. 1. We initially examined the promoter methylation status of miR-126 in five CRC cells. MSP analysis showed extensive methylation of the miR-126 promoter in all five CRC control (BSA-treated) cell lines (Fig. 1). We treated the same panel of CRC cells with 100 μM of each EPA, DHA, and LA. Our results showed that, among all three PUFAs, only DHA completely demethylated the promoter of miR-126 in HCT116 and Caco2 cell lines as compared to the control BSA only-treated cells (Fig. 1). Notably, there was no difference in promoter methylation for miR-126 in SW742, LS180, and HT29/219 cells after PUFA treatment compared with control BSA only-treated cells (Fig. 1).

### PUFA exposure influences gene expression of miR-126 in cultured cells

**Fig. 2:**
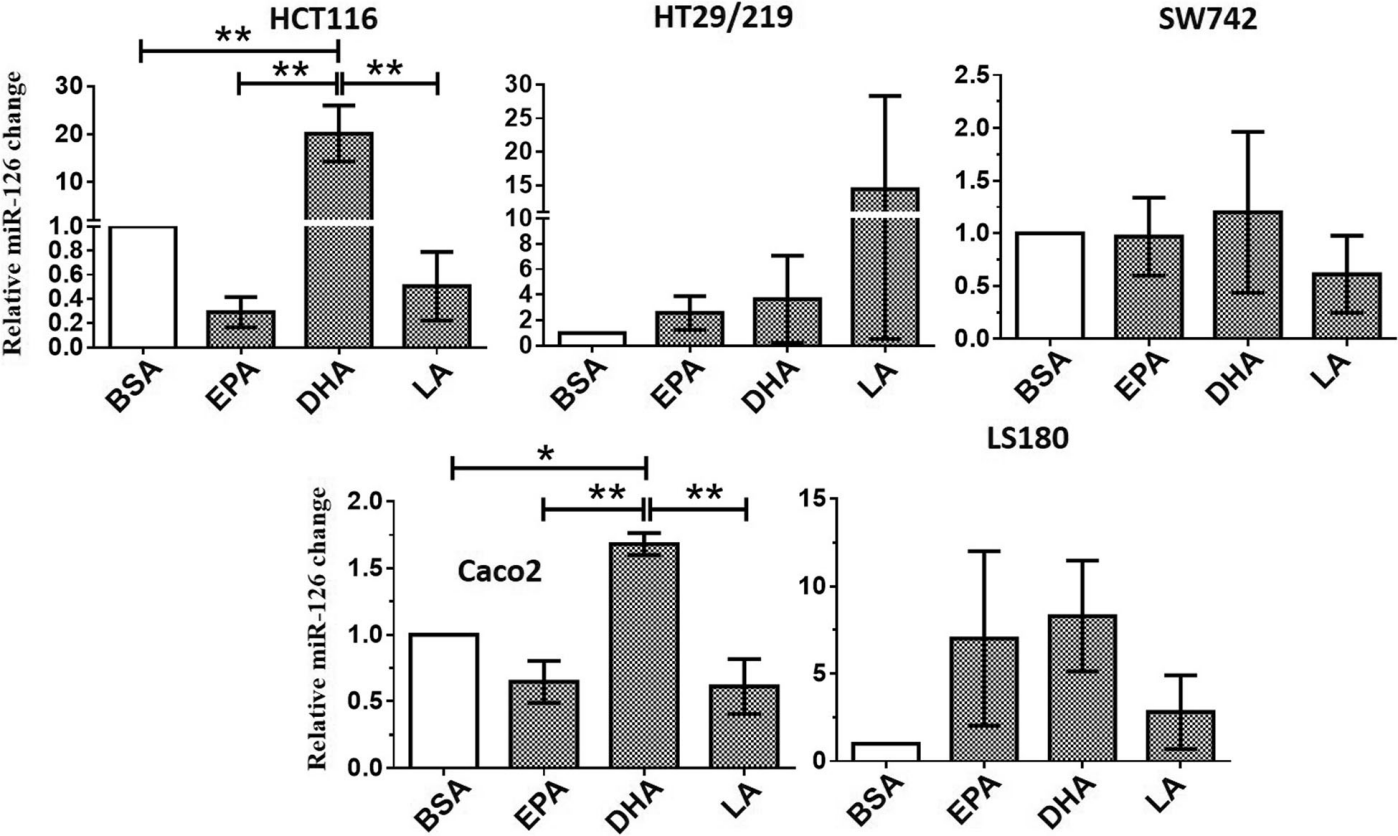
Comparison of the relative expression of miR-126 in PUFA-treated and control cells, measured by quantitative real-time-PCR. Expression of miR-126 was normalized to U6 snRNA. BSA-treated cells were used as a control and expressions in all other PUFA-treated cells were expressed as an *n*-fold difference relative to controls (BSA). Mean values ± SD of the three experiments are given. Bars marked with an asterisk are significantly different as verified by Tukey’s honestly significant difference multiple comparison test (*p* < 0·05). EPA, eicosapentaenoic acid; LA, linoleic acid; DHA, docosahexaenoic acid; BSA, bovine serum albumin

**Fig. 3:**
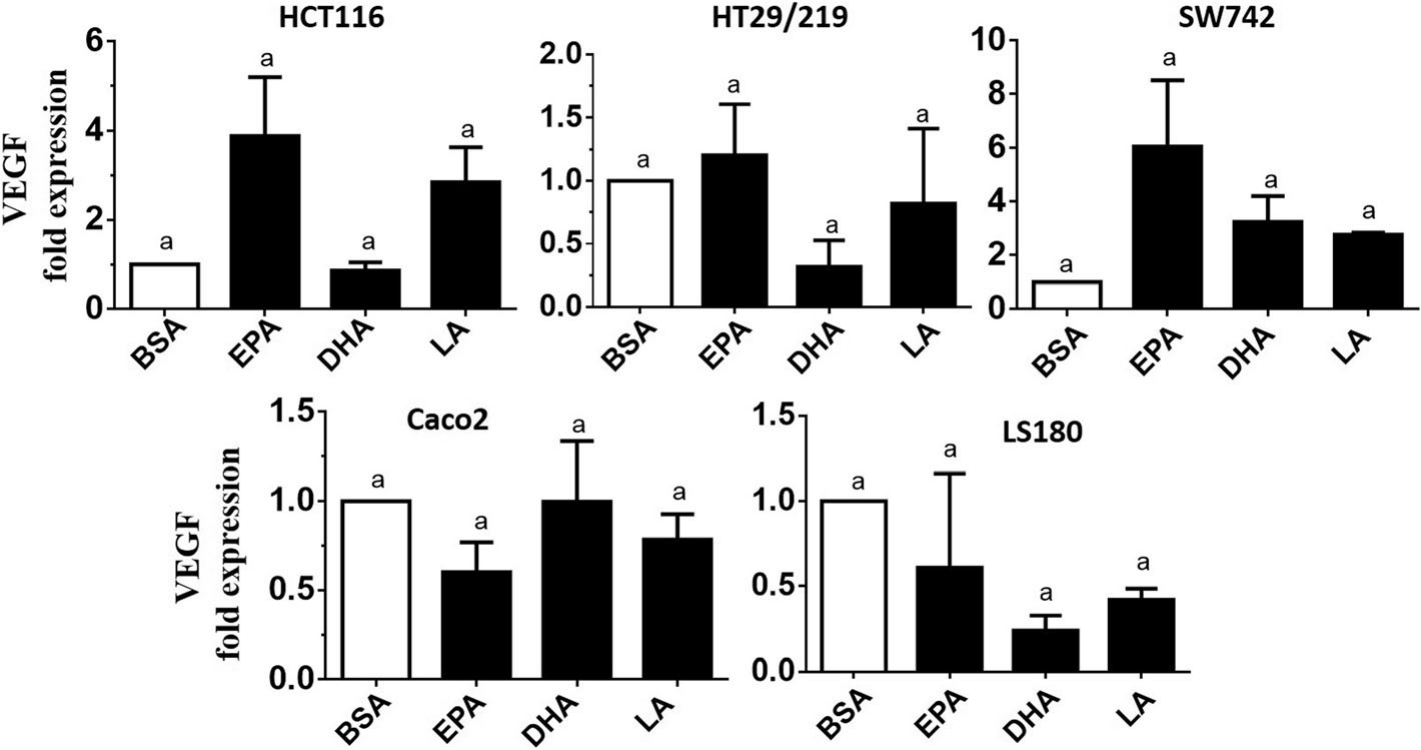
Comparison of the relative expression of VEGF in PUFA-treated and control cells, measured by quantitative real-time-PCR. Expression of VEGF was normalized to GAPDH. BSA-treated cells were used as a control and expressions in all other PUFA-treated cells were expressed as an *n*-fold difference relative to controls (BSA). Mean values ± SD of the three experiments are given, and data are verified by Tukey’s honestly significant difference multiple comparison test (*p* > 0·05). EPA, eicosapentaenoic acid; LA, linoleic acid; DHA, docosahexaenoic acid; BSA, bovine serum albumin

Aiming to verify the influence of EPA, DHA, and LA on miR-126 and VEGF gene expression in CRC cells, we measured the expression level of miR-126 and VEGF by quantitative real-time-PCR in five CRC cell lines (HCT116, HT29/219, SW742, Caco2, and LS180). Stimulation experiments were carried out for 24 h using PUFA in the 100 μM concentration. The relative expression levels of miR-126 are shown in Fig. 2. As shown in Fig. 2, the stimulation of 100 μM DHA significantly upregulated miR-126 expression level by 20.1-fold and 1.68-fold in HCT116 and Caco2 cells, respectively, compared to the BSA-treated control cells (*p* < 0.05). Moreover, in the HCT116 cell line, the miR-126 level was significantly upregulated by DHA by 69-fold and 40-fold in comparison to the EPA and LA-treated cells, respectively (*p* < 0.05). For Caco2 cells, the expression level of miR-126 was significantly upregulated by DHA by 2.6-fold and 2.75-fold compared to the EPA and LA-treated cells, respectively (*p* < 0.05). Also, in this case, DHA showed higher effectiveness than EPA and LA. Furthermore, we found that PUFAs had no significant effects on miR-126 transcripts in HT29/219, SW742, and LS180 cells (*p* > 0.05) (Fig. 2). These results demonstrated that the enhanced expression level of miR-126 was observed only in DHA-treated demethylated HCT116 and Caco2 cells (Fig. 2). Therefore, methylation may result in silencing of miR-126 in these cell lines. However, we found no significant change in VEGF transcript level in five CRC cell lines as verified by real-time PCR (Fig. 3). Based on these results, we hypothesized that miR-126 may target VEGF at the post-transcriptional level. Due to the overexpression of miR-126 in DHA-treated HCT116 and Caco2 cells, we selected these cell lines to verify our hypothesis.

### The effects of PUFA on the protein expression level of VEGF in CRC cell lines

**Fig. 4:**
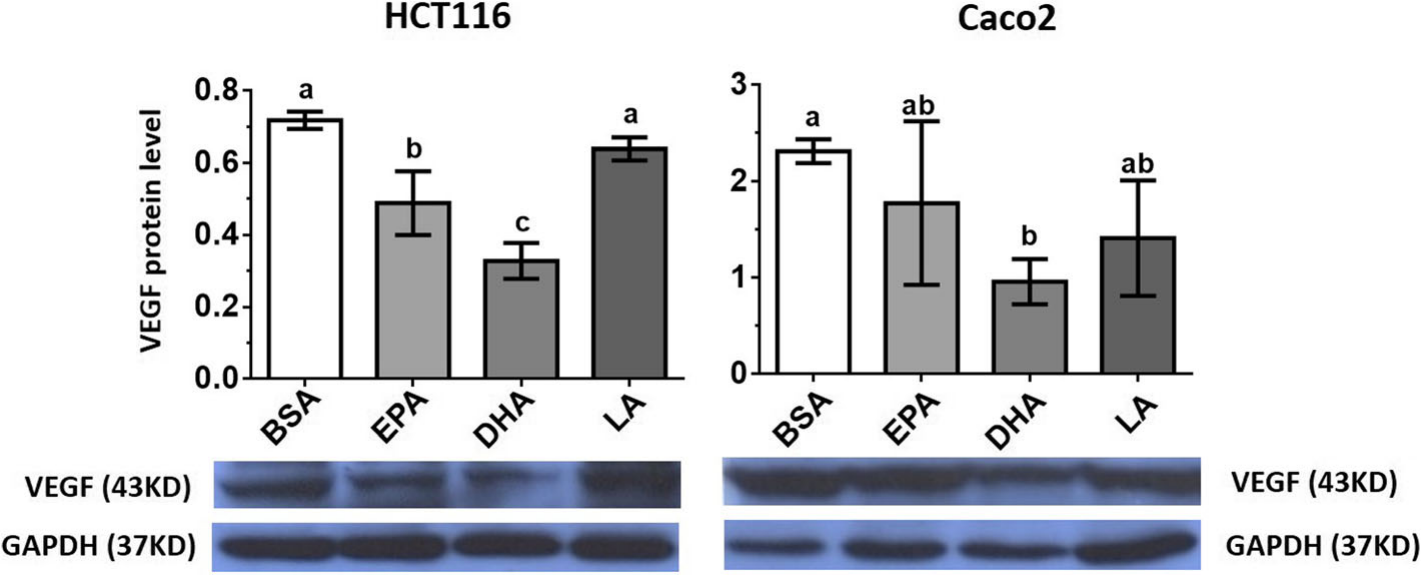
The effect of pure PUFAs on VEGF protein expression level was determined by western blotting analysis in HCT116 and Caco2 cell lines. Mean values ± SD of the three experiments are given. Bars marked with letters are significantly different as verified by Tukey’s honestly significant difference multiple comparison test. EPA, eicosapentaenoic acid; LA, linoleic acid; DHA, docosahexaenoic acid; BSA, bovine serum albumin

Our results showed that VEGF protein expression was more effectively suppressed in DHA-treated HCT116 and Caco2 demethylated cells (*p* < 0.05) (Fig. 4). Both EPA and DHA reduced the production of VEGF in HCT116 cells (reduction: EPA 32%, DHA 54%), but in both HCT116 and Caco2 cell lines, DHA demonstrated higher efficacy than EPA and LA (Fig. 4). However, the upregulation of miR-126 and downregulation of VEGF protein level were effectively observed in the DHA-treated demethylated HCT116 and Caco2 cells (Fig. 4). Our results imply that the suppressed VEGF protein level in HCT116 and Caco2 cells may be partly due to the overexpression of miR-126 caused by DNA demethylation in these cell lines.

## Discussion

In the current study, we indicated that DHA is able to reduce methylation of miR-126 and increase miR-126 gene expression as well as reduce VEGF protein level in HCT116 and Caco2 colorectal cancer cell lines. miR-126 is the mastermind of angiogenesis and metastasis processes in colorectal carcinogenesis that is also known as angiomiRs and metastamiRs [25, 26, 31, 39]. Moreover, recent studies have shown downregulated miR-126 in CRC patients and that DNA methylation results in the epigenetic silencing of miR-126 in colorectal cancer [31, 33, 40]. We found extensive methylation of the miR-126 promoter in all five CRC control (BSA-treated) cell lines (Fig. 1). The identification of epigenetic modifiers and demethylating agents crucial for CRC therapy, as miR-126 gene expression in these tumors, has been related with early detection, therapeutic target, and also a prediction of metastatic CRC patients [39]. Dietary *n*-3 PUFAs seem to be ideal candidates, as they have many beneficial effects and able to modulate the epigenome and reduce tumor growth in both human and rat models [12, 15, 41]. In the present study, we demonstrate for the first time that the 24-h treatment of 100 μM of DHA is able to reduce promoter methylation of miR-126 in only HCT116 and Caco2 cell lines in comparison to control BSA only-treated cells (Fig. 1). Our results show that the five CRC cell lines probably reflect differences in miR-126 promoter methylation in response to PUFA treatment. The demethylating effect of DHA observed by us in Caco2 cells confirms the finding of our previous study obtained in the same colorectal cancer cells [34]. The rationale for using 100 μM concentration as the selected dose was that it is below or within the reported circulating fatty acid range [42]. The rationale for using a 24-h stimulation was that epigenetic responses to fatty acids and lipoproteins were observed in macrophages and cancerous cells cultured in vitro [42–46]. Previous studies reported that PUFA supplementation, namely DHA, at 100 μM for 24 h, may induce cytotoxic effects to cancer cells, altering the normal cellular metabolic pathways and making a possible bias to the final effects/results [47]. However, cancer cells differ from normal cells in their sensitivity to PUFA, and the response to PUFA may be influenced by cell culture conditions including cell culture media, cell plating density, and also tumorigenic status [48]. Moreover, our results indicate that PUFA treatments did not result in changes in the promoter methylation status of miR-126 in HT29/219, SW742, and LS180 cell lines (Fig. 1). However, the five colorectal cancer cell lines investigated in this study varied in appearance, genetic heterogeneity, and also epimarker profiles [49]. HCT116 is negative chromosomal instable (CIN-), but other CRC cell lines including Caco2 and HT29/219 are positive chromosomal instable (CIN+) [49]. Our results are in line with those of others indicated that the ability of PUFAs to modulate epigenetic alterations might be tissue-specific or due to the difference in sensitivity to epigenetic modulators in different cell lines or might relate to fatty acids differences in β-oxidation [46, 50]. Also, we found that miR-126-reduced DNA methylation was accompanied by significantly enhanced gene expression of miR-126 in DHA-treated HCT-116 and Caco2 cells (*p* < 0.05) (Fig. 2). It has been suggested that a high-fat diet modulates expression of miRNAs, and miRNAs could modify DNA methylation through inhibition activity of DNA methyltransferases (DNMTs) [51, 52]. Interestingly, our western blotting analysis demonstrated that significant cell-specific differences in protein expression level of VEGF, a well-known target of miR-126, were also observed effectively only in DHA-treated demethylated HCT116 and Caco2 cell lines (Fig. 4). VEGF is a potent angiogenic factor with a well-defined role in the formation of new blood vessels in colorectal cancer [53]. These results imply that DNA methylation resulted in the silencing of miR-126 in HCT116 and Caco2 cells, and restoration of miR-126 by DHA may be partly responsible for the low VEGF protein level in HCT116 and Caco2 cells. We have found previously that the HCT116 and Caco2 cells significantly expressed high levels of DNMTs compared to other CRC cell lines [54]. Moreover, we previously verified that *n*-3 and *n*-6 PUFAs significantly downregulated DNMTs in HCT116 and Caco2 cells [34]. Both EPA and DHA reduce VEGF protein expression level in HCT116 cells, but they do not show similar effectiveness, with DHA being much more efficient than EPA and LA not only in reducing the promoter methylation of miR-126 but also in increasing level of miR-126 and decreasing level of VEGF protein expression in both HCT116 and Caco2 cell lines. Experimental and clinical studies have indicated that DHA can sensitize colorectal cancer cells to antineoplastic factors and enhance tumor responsiveness to chemotherapeutic agents [55]. Furthermore, in vitro studies using HepG2 human hepatoma cells demonstrated that DHA significantly enhanced antioxidant enzymes and also enhanced cancer cells susceptibility to H_2_O_2_ [56, 57]. From the mechanistic viewpoint, DHA influences the dynamics of protein localized in membrane lipid rafts, modulates the activity of membrane transporters and cell signaling pathways, and consequently affects cell behavior [58]. Moreover, it has been suggested that DHA incorporation into phospholipids bilayer is tissue-specific and comprising about 50% of the membrane’s total acyl chain [59]. DHA is readily incorporated into cell membrane micro-domains and modulates miRNAs expression [17]. Another possible mechanism is that methyl groups from S-adenosyl methionine (SAM) are required for the conversion of phosphatidylethanolamine-DHA (PE-DHA) to phosphatidylcholine-DHA (PC- DHA). Upon tissue and cellular DHA is lacking, there is less PE-DHA and the resulting excess in methyl groups will be available for other transmethylation reactions of DNA by DNMTs [60]. Based on this mechanism, in HCT116 and Caco2 cells, DHA can alter promoter DNA methylation of miR-126 by changing the activity of DNMTs. PUFA also influence DNA methylation by interfering with membrane-associated cellular signal transduction including the Ras signaling pathway. Accordingly, the activation of Ras signaling induces DNMT1 gene expression and excess of DNMT1 levels may target certain genes for hypermethylation [61–63]. Also, it has been suggested that fatty acids could bind to intracellular transcription factors such as peroxisome proliferator-activated receptors (PPARs) and regulate gene repression [64]. Overall, the upregulation of miR-126 and reduced levels of VEGF were observed in the DHA-exposed demethylated HCT116 and Caco2 cells (Figs. 2 and 4). Therefore, these results demonstrate that the molecular features and epigenetic differences of colorectal cancer cells can contribute to the observed variations in response to PUFA exposure. Our results show that there is some selectivity to respond effectively to PUFAs by promoting the miR-126 promoter demethylation in different colorectal cancer cell lines. While the precise molecular mechanistic basis by which PUFAs modulate cell- and gene-specific methylation is not clear, previous studies have reported similar effects of demethylating agents on CRC cell lines and suggested that cell type-specific resistance mechanisms may be involved [50, 65, 66]. However, mounting evidence suggests that colorectal cancer comprises a group of molecularly heterogeneous diseases that undergo a variety of clinical courses and possess diverse therapeutic responses [67, 68].

Although the beneficiary effects of dietary PUFA on reducing the risk of cancer is widely acknowledged, the multiple possible mechanisms are only starting to be resolved. Characterizing the molecular mechanism(s) by which *n*-3 PUFAs suppress tumor growth will provide an opportunity to develop personalized diets for cancer control. We believe that our present study has some limitations. First of all, the MSP technique used in this study is not a quantitative assay and could give false positive or false negative results. Furthermore, our study was limited to only miR-126 methylation CpG sites; so, further studies are required to determine the DNA methylation status of miRNAs using quantitative assays including high-throughput deep Pyro-sequencing. Nevertheless, by linking PUFA with DNA methylation, our study provides some insight regarding epigenetic modification by PUFA. In order to get conclusive results, further in vivo studies, both in animals and in humans, are needed, and these findings would be useful for dietary interventions in cancer.

## Conclusions

Overall, our results demonstrate that PUFA, namely DHA, can alter the miR-126 promoter DNA methylation as well as the VEGF protein expression in a cell type-specific manner. DHA (*n*-3 PUFA) is more effective than EPA (*n*-3 PUFA) and LA (*n*-6 PUFA) in attenuating promoter DNA methylation of miR-126 as well as VEGF protein level in HCT116 and Caco2 colorectal cancer cells. Our study offers new insights into the epigenetic mechanisms by which PUFA influence gene expression regulation in colorectal cancer cells.

## Abbreviations

3′UTR: Three prime untranslated region
CIN: Chromosomal instable
CRC: Colorectal cancer
DHA: Docosahexaenoic acid
DNMT: DNA methyltransferase
EGFL7: Epidermal growth factor-like domain 7 gene
EPA: Eicosapentaenoic acid
LA: Linoleic acid
miR: MicroRNA
MSP: Methylation-specific PCR
PUFA: Polyunsaturated fatty acid
VEGF: Vascular endothelial growth factor
